# ﻿Endemic taxon or environmentally induced colour morph? Genetic insights into the insular subspecies of *Platycepsnajadum* (Serpentes, Colubridae) from Kalymnos, Greece

**DOI:** 10.3897/zookeys.1244.152290

**Published:** 2025-07-04

**Authors:** Daniel Jablonski, Elias Tzoras

**Affiliations:** 1 Department of Zoology, Comenius University in Bratislava, Ilkovičova 6, Mlynská dolina, 842 15, Bratislava, Slovakia Comenius University in Bratislava Bratislava Slovakia; 2 Patras 264 42, Achaia, Greece Unaffiliated Patras Greece

**Keywords:** Aegean, Dodecanese, endemism, melanism, validity, taxonomy

## Abstract

Phenotypic variation, particularly in colouration, is well documented in snake populations across the Balkans, with insular populations often exhibiting distinct morphological traits. Historically, these differences have been used to define subspecies or even new species. However, many such classifications have been revised following genetic analysis. One unresolved taxonomic case involves the subspecies *Platycepsnajadumkalymnensis* (Schneider, 1979), a melanistic taxon endemic to Kalymnos Island, Greece. In this study, we present the first genetic data (both mitochondrial and nuclear) for *P.n.kalymnensis*, comparing it with populations from the Balkan–Anatolian range to clarify its evolutionary status. Mitochondrial DNA analysis reveales that *P.n.kalymnensis* clusters closely with the Balkan and Anatolian populations, exhibiting low levels of genetic differentiation. However, nuclear markers reveal distinct allelic variations, suggesting a degree of evolutionary independence, likely influenced by the island’s long-term specific environmental conditions. Our results support the continued recognition of *P.n.kalymnensis* as a distinct subspecies, though further studies using high-resolution genomic data are needed to better understand its evolutionary trajectory.

## ﻿Introduction

Variation in phenotypes, particularly colour morphs across different populations, is well documented in snakes from both mainland and island regions of the Balkans (Jablonski et al. 2017, 2023). Such phenotypic differences have often been taxonomically evaluated over two past centuries, especially in populations from the islands of the Aegean Sea (Dimitropoulos 1986) and have sometimes been described as distinct species or subspecies (see Mertens and Wermuth 1960). Due to the isolation of the islands, taxonomists in the past believed these populations were unique and considered them evolutionarily independent. However, they lacked genetic information, which has led to numerous of these descriptions to be synonymized after genetic data had become available (e.g. Utiger and Schätti 2004; Asztalos et al. 2021).

Several taxa inhabiting the islands of the Balkan region have been reported to exhibit local phenotypic variations, including erythristic individuals of Macrovipera (lebetinus) schweizeri (Werner, 1935), striped phenotypes of *Zamenissitula* (Linnaeus, 1758), melanistic individuals of *Eirenismodestussemimaculatus* (Boettger, 1876), Vipera (ammodytes) meridionalis Boulenger, 1903 or *Dolichophiscaspius* (Gmelin, 1789), as well as albinism in *Eryxjaculusturcicus* (Olivier, 1801) (Schweizer 1935; Clark 1968; Cattaneo 2010; Itescu et al. 2015; Kalaentzis et al. 2018; Kalogiannis 2021). A well-documented example is the variation in Aegean populations currently classified under *Natrixnatrixmoreotica* (Bedriaga, 1882), which has historically led to the description of several names and contributed to biogeographical confusion (Jablonski et al. 2023).

**Figure 1.**
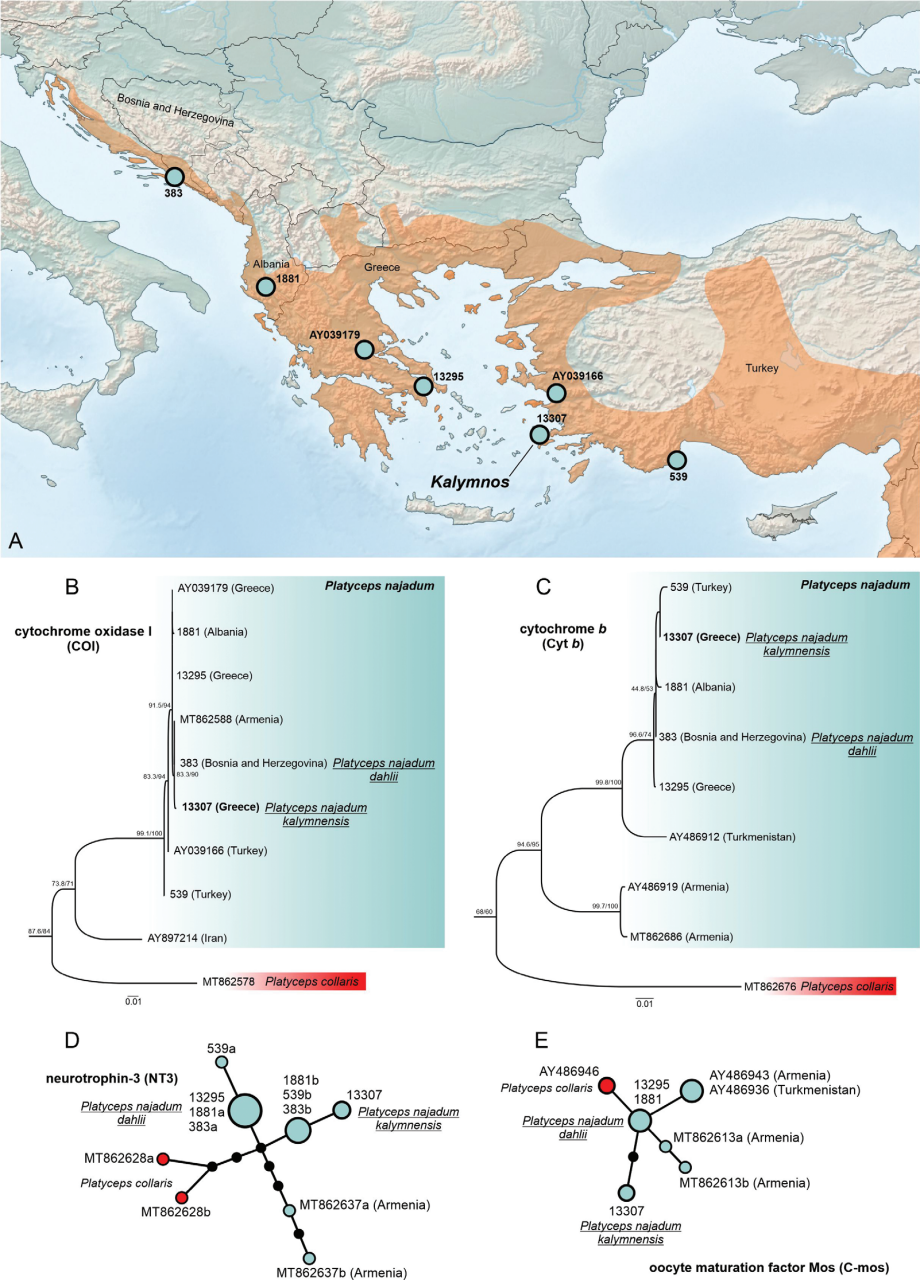
**A.** Southeastern Europe and western Asia highlighting distribution of *Platycepsnajadum* (shaded in orange); sampling locations of specimens studied marked with circles labelled with a specimen ID; **B.** Phylogenetic tree showing the relationship of *P.najadum* specimens based on COI sequences; *Platycepsnajadumkalymnensis* (specimen 13307) indicated, and *Platycepscollaris* (in red) is used as the outgroup; **C.** Phylogenetic tree constructed using cyt *b* sequences, showing placement of *P.n.kalymnensis* (13307); support values are indicated on the branches; **D.** Haplotype network illustrating the genetic variation among *P.najadum* specimens based on NT3 sequences; circles represent different haplotypes, with black dots indicating mutational steps, and the red node represents *P.collaris*; **E.** Haplotype network showing genetic relationships among specimens using C-mos sequences; the red node represents *P.collaris*, while different alleles of *P.najadum* are indicated. Different alleles of a single heterozygous sequence are coded with ‘a’ and ‘b’, homozygous sequences are without this label. Refer to Table S2 for locality details and sample codes used in the tree and networks. The geographic distribution of the species in the study area is highlighted in orange.

This issue also applies to Dahl’s whip snake, *Platycepsnajadum* (Eichwald, 1831), a species of the family Colubridae. The type locality of *P.najadum* is in Baku, Azerbaijan. However, the species has a broad Eurasian distribution, spanning from Turkmenistan and Iran through the southern Caucasus Mountains, part of the Levant, Asia Minor, and the southern Balkans, including numerous islands in the Adriatic, Ionian, and Aegean Seas (Sindaco et al. 2013; Fig. 1A). Its taxonomy remains unsettled, with five currently recognized subspecies depending on the author: *Platycepsn.najadum* (Eichwald, 1831), *P.n.dahlii* (Schinz, 1833) with the type locality in “Dalmatia” on the Adriatic coast, *P.n.kalymnensis* (Schneider, 1979) with the type locality on Kalymnos island, Greece, *P.n.atayevi* (Tuniyev & Shammakov, 1993) with the type locality in western Kopet-Dag, Turkmenistan, and *P.n.albitemporalis* (Darevsky & Orlov, 1994) with the type locality near Lenkoran in south-eastern Azerbaijan. Throughout its range, *P.najadum* typically exhibits a uniform beige to olive-green base colour with a series of dark spots with white borders on both sides of the neck. However, darker individuals also occur throughout its distribution range, for example in the Caucasian part of the Black Sea coast (Schätti et al. 2005; Tuniyev et al. 2019).

**Figure 2.**
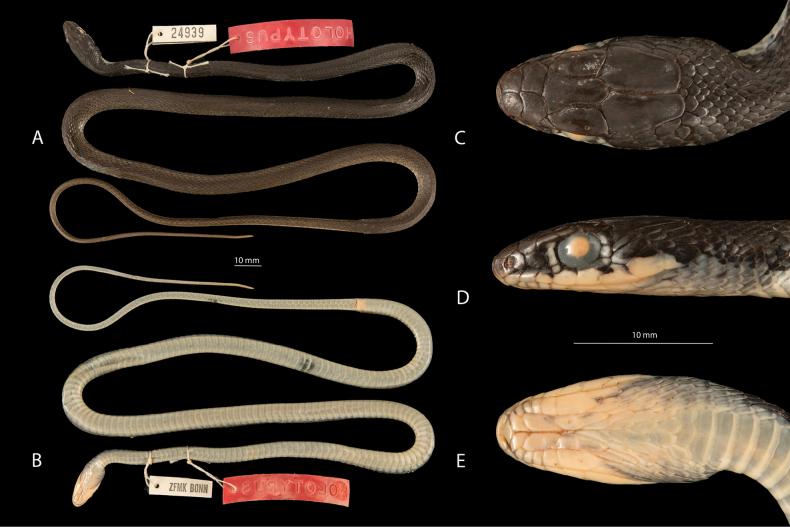
Holotype of *Platycepsnajadumkalymnensis* (ZFMK 24939; female) collected by Bert Schneider on 2 August 1977. **A.** Dorsal view showing body length and scale arrangement; **B.** Lateral view illustrating elongate body and head shape; **C.** Close-up of head in dorsal view, highlighting scalation and colouration pattern; **D.** Lateral view of head, emphasizing eye and temporal region; **E.** Ventral view of head, showing snout and other key morphological features. Photos by Flecks Morris. Scale bar: 10 mm (**B**).

Uniquely, partially black-coloured populations are known only from the eastern Aegean, particularly on Kalymnos Island in Greece (Schneider 1979, 1983; Cattaneo 2005). These snakes have been described as the subspecies *Colubernajadumkalymnensis* Schneider, 1979. This taxon represents yet another description based solely on unique colouration, though its taxonomic validity has never been genetically confirmed and remains doubtful (Sindaco et al. 2013). The description is based on two examined, almost fully black-coloured specimens (see the ventral part of the holotype in Fig. 2) from Kalymnos, specifically from “SW-Kalymnos, südl. des Klosters Agios Ekaterinis, ca. 150 m NN” (36.9362°N, 26.9490°E, 173 m). In his original publication, Schneider (1979) stated that apart from melanism in adult individuals, there were no significant differences from the closest Turkish populations, except for the low number of dorsal scale rows (17) counted in the paratype. He also noted that melanism appeared only in adult individuals, with juveniles exhibiting the normal base colouration, which darkens with age (see iNaturalist observation no. 278759073). Thus, as suggested by multiple authors (Engelmann et al. 1986; Foufopoulos 1997; Cattaneo 2005), *P.n.kalymnensis* may represent an island form adapted to local environmental conditions rather than a taxon with long-term isolated evolution.

To resolve this nearly half-century-old taxonomic question, we analysed, for the first time, genetic data from the *P.najadum* population on Kalymnos to provide new evidence regarding its taxonomic status.

## ﻿Methods

Tissue samples used for this study were mostly collected from snakes found killed. The only available sample from Kalymnos was a dry mummy of dark, partly melanistic colouration, collected by us near Arginonta, Kalymnos, Greece (NHMC 80.3.132.20; sample 13307; 37.0033°N, 26.9975°E, 305 m a.s.l.; Suppl. material 1: fig. S1). The dataset also included populations from the western Balkans, particularly from Dalmatia, represented here by a road-kill sample (383) from the Mediterranean part of Bosnia and Herzegovina affiliated with the subspecies *P.n.dahlii*, a taxon geographically closely related to *P.n.kalymnensis*. Available samples of *P.najadum* were preserved in 96% ethanol. DNA was extracted using the E.Z.N.A.® Tissue DNA Kit (Omega Bio-tek, Inc., Norcross, GA, USA). Sequences of two mitochondrial (mtDNA) and two nuclear (nuDNA) genes were targeted: the mitochondrial protein-coding segments cytochrome oxidase I (COI) and cytochrome b (cyt *b*), and the nuclear protein-coding genes neurotrophin-3 (NT3) and oocyte maturation factor Mos (C-mos). For PCR, we used Red Taq 2X Master Mix with 2 mM MgCl_2_. The primers and PCR conditions used for each marker are presented in Suppl. material 1: table S1. The same primers were also used for sequencing. PCR products were purified with ExoSAP-IT PCR Product Cleanup Reagent (Applied Biosystems, Foster City, CA, USA), following the manufacturer’s protocol. Sequencing was performed by Macrogen Europe Inc. (Amsterdam, Netherlands; https://www.macrogen-europe.com/).

We performed a BLAST search in GenBank (https://blast.ncbi.nlm.nih.gov/Blast.cgi) to confirm that the targeted loci and species were amplified. The translation of protein-coding sequences into amino acids was checked using DnaSP 6.00, confirming the absence of stop codons. New sequences were combined with previously published data from GenBank (Suppl. material 1: table S2). Sequence alignment and manual editing were performed using Seaview 5 (Gouy et al. 2021). The final alignments were analyzed using maximum likelihood (ML) in IQ-TREE (Nguyen et al. 2015; Trifinopoulos et al. 2016) under default settings. The best-fit model for the single-gene dataset was selected using ModelFinder (Kalyaanamoorthy et al. 2017) implemented in IQ-TREE. We created two separate mtDNA datasets: one including available COI gene sequences (642 bp) and the other including cyt *b* sequences (1,134 bp) of *P.najadum* and other selected species of the genus *Platyceps* (Suppl. material 1: table S2), with *Spalerosophisdiadema* as an outgroup. Branch support was evaluated using the Shimodaira–Hasegawa-like approximate likelihood ratio test (SH-aLRT; Guindon et al. 2010) and the Ultrafast Bootstrap approximation method (UFBoot; Minh et al. 2013), both performed with 1,000 replicates. Nodes with values of 95 and above were considered strongly supported, while nodes with values between 70 and 90 were considered well supported. DnaSP 6.00 (Rozas et al. 2017) was used to estimate nucleotide diversity (π) and uncorrected *p*-distances in the mitochondrial datasets. The newly generated sequences of *P.najadum* were deposited in GenBank (Suppl. material 1: table S2).

To assess if the mitochondrial DNA results correspond with nuclear data, we analysed selected *P.najadum* samples across the range using allele network analysis on the nuclear DNA markers C-mos and NT3. We used previously generated sequences from GenBank (Suppl. material 1: table S2) and newly obtained sequences from *P.najadum* populations, including the endemic *P.n.kalymnensis* from Kalymnos and the geographically close taxon *P.n.dahlii* from Dalmatia. The resulting sequence alignments (499 bp for NT3, 567 bp for C-mos) were analysed using PHASE 2.1.1 (Stephens et al. 2001) for heterozygous site resolution, with input data prepared in SeqPHASE (Flot 2010). PHASE was run under default settings, with the probability threshold set to 0.9. Allele networks of both nuclear markers were visualized in PopArt (Leigh and Bryant 2015) using the implemented parsimony network algorithm of TCS (Clement et al. 2000) with a 95% connection limit.

## ﻿Results

The phylogenetic analyses based on mitochondrial (COI and cyt *b*) and nuclear (NT3, C-mos) markers reveal genetic structuring in *Platycepsnajadum* (Fig. 1). The phylogenetic tree inferred from COI (Fig. 1B) shows that *P.najadumkalymnensis* clusters within the species lineage, which includes sequences from the western (383, 1881) and southern Balkans (AY039179, 13295), western Anatolia (AY039166, 539), and, surprisingly, Armenia (MT862588 from Šmíd et al. 2021). This lineage is sister to a sequence from Iran, with an uncorrected *p*-distance of 8.7%. The bootstrap values support its position within this lineage, and the genetic distinction from other populations suggests only regional differentiation, as indicated by low nucleotide diversity (π = 0.4%).

Similarly, the cytochrome *b* tree (Fig. 1C) places *P.najadumkalymnensis* within a well-supported lineage, clustering with samples from the Balkans and western Anatolia and showing very shallow genetic differentiation (π = 0.6%). This lineage is sister to a sequence from Turkmenistan (AY486912, related to *P.n.atayevi*; Nagy et al. 2004), with an uncorrected *p*-distance of 2.8%. Both lineages are sister to a lineage representing sequences from Armenia (AY486919, MT862686; Nagy et al. 2004; Šmíd et al. 2021) with a *p*-distance of 8.0%, which differs notably from the COI phylogeny results.

The nuclear NT3 gene (Fig. 1D) shows that the *P.n.kalymnensis* allele differs by a single mutation step from geographically close populations in the western Balkans and Anatolia. However, it exhibits a unique allele indicative of genetic differentiation, which contrasts with the mitochondrial data. Overall, we detected six alleles of *P.najadum* in the analysed NT3 sequences, with the most divergent alleles originating from Armenia (MT862637).

The C-mos gene (Fig. 1E) supports this pattern, with *P.n.kalymnensis* and alleles representing populations from the Balkans forming a distinct allele within *P.najadum*, separated by two mutation steps. The allele from Armenia and Turkmenistan are separated by three (AY486936, AY486943), and four mutation steps (MT862613) from the Kalymnos taxon. Overall, five alleles were detected in the analysed dataset.

## ﻿Discussion

For the first time, we have analysed the DNA of the endemic subspecies *Platycepsnajadumkalymnensis* from the Kalymnos Island, Greece, and compared it with several populations from the Balkan–Anatolian region. This island, part of the Dodecanese Archipelago, has been far less studied herpetologically than other well-known islands such as Milos, Rhodes, Lesbos, or Crete (Müller 1932; Buttle 1995; Broggi 2000; Cattaneo 2005; Bader et al. 2009). Kalymnos is located just 17 km from the Anatolian mainland and approximately 10 km from Kos, another major island in the Dodecanese. With a total area of around 135 km^2^, it is the third most populated island in the archipelago.

Given its relatively small size, it is remarkable that Kalymnos hosts a morphologically distinct population of dark coloured *P.najadum*, the only known partly melanistic colour form of this species in the Aegean region and a rarity within its broader distribution range (e.g. previously reported only from the Greek island of Chios and the Caucasian Black Sea coast; Dimitropoulos 1990; Darevskij and Szczerbak 1993; Tuniyev et al. 2019). Since melanistic populations of different snake species have occasionally been documented on the dry islands of the Aegean (Kalaentzis et al. 2018; Kalogiannis 2021), we initially hypothesized that such colouration in this local population is a response to specific environmental factors on the island, while its genetic composition would align with populations from the Balkans or western Anatolia.

However, our findings only partially support this assumption. While environmental conditions likely play a role, genetic data, especially nuclear DNA, reveal the level of differentiation. Kalymnos is predominantly composed of limestone, with extensive karst formations, caves, and rock walls, distinguishing it from some other islands in the region. The terrain is mountainous and rocky, with limited forested areas and relatively little fertile soil, which affects vegetation. Rainfall is scarce, natural water resources are limited, and the island lacks permanent rivers. The vegetation consists mainly of maquis and phrygana, dominated by drought-resistant shrubs and herbs, creating an overall arid environment. This may influence melanism in snakes inhabiting Mediterranean areas, as such habitats could provide thermal or cryptic advantages for the melanistic phenotype while aiding in predator avoidance (see, for example, the presence of melanism in Mediterranean populations of *Dolichophisjugularis* or *Hierophisviridiflavus*). A similar hypothesis has been proposed for melanistic populations of *Natrixnatrix* (Fănaru et al. 2022) and could also explain the high phenotypic variation with a tendency toward melanism in other Aegean snake populations (Clark 1968; Cattaneo and Grano 2013; Kalaentzis et al. 2018; Kalogiannis 2021; Jablonski et al. 2023). These findings thus open the door for further research on the role of genetic components and morphology in relation to environmental variables that should be tested as working hypothesis.

Our genetic analysis indicates that while the population of *P.n.kalymnensis* shows shallow differentiation in the mitochondrial DNA, suggesting a close relationship with Balkan–Anatolian populations (possibly *P.n.dahlii*), it displays distinct allelic variations in nuclear genes (homozygous for different alleles). This supports an evolutionary and thus taxonomic distinction. Similar results were presented by Šmíd et al. (2021) in the case of the sister species *P.collaris* (Müller, 1878). Considering the island’s isolation, the population’s morphological uniqueness, and our genetic evidence, along with the fact that the subspecies name already exists, we conclude that the taxonomic recognition of this endemic form can be considered valid. However, further research is needed to assess its conservation status.

Notably, this is not the first such case in the region. Recently, Javorčík et al. (2024) suggested, based on both mitochondrial and nuclear data, that *Dolichophisjugulariszinneri* Cattaneo, 2012 from Dodecanese Island of Rhodes, has an evolutionary basis, despite long being considered a dubious taxon. Conversely, some taxonomic designations have not been supported by genetic investigations, such as *Elapherechingeri* Werner, 1932, described from Amorgos Island in the Cyclades (Werner 1932) primarily due to its uniform colouration, which also includes melanism. This taxon was later recognized as an insular variant of the Cycladic endemic *E.quatuorlineatamuenteri* (Bedriaga, 1882) (Cattaneo and Grano 2013; Thanou et al. 2020). The same conclusion was reached in the case of another colubrid snake, *Colubergemonensisgyarosensis* Mertens, 1968, which was found to be an introduced population of *Hierophisviridiflavus* on Gyaros Island, Greece (Mertens 1968; Utiger and Schätti 2004). However, phenotypic diversity in the context of molecular data should be broadly investigated throughout the Aegean, as it may yield new and surprising evidence for other lesser-studied reptiles in the region (e.g. genera *Eirenis*, *Hemorrhois*, or *Telescopus*).

As our results show, the taxonomy and phylogeography of *P.najadum* is poorly investigated considering its morphological diversity (Schätti et al. 2005). To clarify the broader diversity of this species, additional research should focus on the genetic variation using high-resolution genomic approaches combined with more geographically extensive sampling and morphological data.

## ﻿Additional information

### Author ORCIDs

Daniel Jablonski https://orcid.org/0000-0002-5394-0114

Elias Tzoras https://orcid.org/0009-0006-7108-3052

### Data availability

All of the data that support the findings of this study are available in the main text or Supplementary Information.

## Supplementary materials

Supplementary material 1 Additional information Data type pdf Explanation note **table S1.** A list of DNA loci, their corresponding primer sequences, sources, and the specific PCR conditions used for amplifications. **table S2.** Material used for the genetic analysis. **fig. S1.** DNA-investigated specimen of *Platycepsnajadumkalymnensis* (NHMC 80.3.132.20) from Kalymnos Island, Greece. Photo by Daniel Jablonski. https://binary.pensoft.net/file/1367617This dataset is made available under the Open Database License (http://opendatacommons.org/licenses/odbl/1.0/). The Open Database License (ODbL) is a license agreement intended to allow users to freely share, modify, and use this Dataset while maintaining this same freedom for others, provided that the original source and author(s) are credited.Daniel Jablonski, Elias Tzoras

## References

[B1] Asztalos M Ayaz D Bayrakci Y Afsar M Tok CV Kindler C Jablonski D Fritz U (2021) It takes two to tango—phylogeography, taxonomy and hybridization in grass snakes and dice snakes (Serpentes: Natricidae: *Natrixnatrix*, *N.tessellata*). Vertebrate Zoology 71: 813–834. 10.3897/vz.71.e76453

[B2] Bader T Riegler C Grillitsch H (2009) The herpetofauna of the island of Rhodes (Dodecanese, Greece). Herpetozoa (Wien) 21: 147–169.

[B3] Broggi MF (2000) Herpetological notes on the islands of Milos and Sifnos (Cyclades, Greece). Herpetozoa (Wien) 13: 89–93.

[B4] Buttle D (1995) An introduction to the amphibians and reptiles of the Greek islands. Reptilian Magazine 3: 15–25.

[B5] Cattaneo A (2005) Nuovo contributo alla conoscenza dell’erpetofauna dell’isola egea di Kalymnos (Sporadi meridionali). Bollettino del Museo civico di storia naturale di Venezia 56: 153–163.

[B6] Cattaneo A (2010) Note eco-morfologiche su alcune specie ofidiche egee,con particolare riferimento alle popolazioni delle Cicladi centro-orientali (Reptilia). Naturalista siciliano S. IV, XXXIV (3–4): 319–350.

[B7] Cattaneo A Grano M (2013) The Aegean populations of *Elaphequatuorlineata* (Lacépède, 1789): A morpho-ecological examination. In: Schulz KD (Ed.) Old World Ratsnakes.A Collection of Papers. Bushmaster Publications, Berg, Switzerland, 269–288.

[B8] Clark RJ (1968) Außergewöhnliche Färbung und Zeichnung bei *Viperaammodytesmeridionalis* von griechischen Inseln. Salamandra (Frankfurt) 4(2/3): 69–72.

[B9] Clement M Posada D Crandall KA (2000) TCS: A computer program to estimate gene genealogies. Molecular Ecology 9(10): 1657–1659. 10.1046/j.1365-294x.2000.01020.x 11050560

[B10] Darevskij IS Szczerbak NN (1993) *Colubernajadum* (Eichwald, 1831) – Schlanknatter. In: Böhme W (Ed.) Handbuch der Reptilien und Amphibien Europas.Band 3/I. Aula-Verlag, Wiesbaden, 131–144.

[B11] Dimitropoulos A (1986) Some notes on the colour and pattern variation of the Greek snake fauna in relation to geographic distribution. Biologia Gallo-Hellenica 12: 463–471.

[B12] Dimitropoulos A (1990) A melanistic whip-snake, *Colubernajadum* (Eichwald), from the Greek island of Chios, eastern Aegean. Annales Musei Goulandris 8: 241–243.

[B13] Engelmann WE Fritzsche J Günther R Obst FJ (1986) Lurche und Kriechtiere Europas. Enke, Stuttgart, 420 pp.

[B14] Fănaru G Telea AE Gherghel I Melenciuc R (2022) Melanism in the grass snake *Natrixnatrix* (Linnaeus, 1758) from the Danube Delta Biosphere Reserve, Romania. Herpetozoa (Wien) 35: 257–263. 10.3897/herpetozoa.35.e85310

[B15] Flot JF (2010) SeqPhase: A web tool for interconverting phase input/output files and fasta sequence alignments. Molecular Ecology Resources 10(1): 162166. 10.1111/j.1755-0998.2009.02732.x 21565002

[B16] Foufopoulos J (1997) The reptile fauna of the northern Dodecanese (Aegean Islands, Greece). Herpetozoa (Wien) 10(1/2): 3–12.

[B17] Gouy M Tannier E Comte N Parsons DP (2021) Seaview v.5: A multiplatform software for multiple sequence alignment, molecular phylogenetic analyses, and tree reconciliation. Methods in Molecular Biology (Clifton, N.J.) 2231: 241–260. 10.1007/978-1-0716-1036-7_15 33289897

[B18] Guindon S Dufayard JF Lefort V Anisimova M Hordijk W Gascuel O (2010) New algorithms and methods to estimate maximum-likelihood phylogenies: Assessing the performance of PhyML 3.0. Systematic Biology 59(3): 307–321. 10.1093/sysbio/syq010 20525638

[B19] Itescu Y Savyon G Slavenko A (2015) *Eryxjaculus* (Sand boa). Albinism. Herpetological Review 46(1): 103–104.

[B20] Jablonski D Szabolcs M Simović A Mizsei E (2017) Color and pattern variation of the Balkan whip snake, *Hierophisgemonensis* (Laurenti, 1768). Turkish Journal of Zoology 41: 363–369. 10.3906/zoo-1606-15

[B21] Jablonski D Tzoras E Panagiotopoulos A Asztalos M Fritz U (2023) Genotyping the phenotypic diversity in Aegean *Natrixnatrixmoreotica* (Bedriaga, 1882) (Reptilia, Serpentes, Natricidae). ZooKeys 1169: 87–94. 10.3897/zookeys.1169.104594 38322271 PMC10846685

[B22] Javorčík A Strachinis I Thanou E Kornilios P Avcı A Üzüm N Olgun K Ilgaz Ç Kumlutaş Y Lymberakis P Nagy ZT Jablonski D (2024) Phylogeography of *Dolichophis* populations in the Aegean region (Squamata: Colubridae) with taxonomic remarks. Diversity 16(3): 184. 10.3390/d16030184

[B23] Kalaentzis K Kazilas C Strachinis I (2018) Two cases of melanism in ring-headed dwarf snake *Eirenismodestus* (Martin, 1838) (Serpentes: Colubridae) from Kastellorizo, Greece. Herpetology Notes 11: 175–178.

[B24] Kalogiannis S (2021) Cases of melanism in *Dolichophiscaspius* (Gmelin, 1789) (Squamata: Colubridae) from Greece and a new distribution record. Parnassiana Archives 9: 19–22.

[B25] Kalyaanamoorthy S Minh BQ Wong TKF von Haeseler A Jermiin LS (2017) ModelFinder: Fast model selection for accurate phylogenetic estimates. Nature Methods 14(6): 587–589. 10.1038/nmeth.4285 28481363 PMC5453245

[B26] Leigh JW Bryant D (2015) Popart: Full-feature software for haplotype network construction. Methods in Ecology and Evolution 6(9): 1110–1116. 10.1111/2041-210X.12410

[B27] Mertens R (1968) Eine schwarze Zornnatter von den Cycladen: *Colubergemonensisgyarosensis* n. subsp. Senckenbergiana Biologica 46(1): 5–9.

[B28] Mertens R Wermuth H (1960) Die Amphibien und Reptilien Europas (Dritte Liste, nach dem Stand vom 1. Januar 1960). Verlag Waldemar Kramer, Frankfurt am Main, 264 pp.

[B29] Minh BQ Nguyen MAT von Haeseler A (2013) Ultrafast approximation for phylogenetic bootstrap. Molecular Biology and Evolution 30(5): 1188–1195. 10.1093/molbev/mst024 23418397 PMC3670741

[B30] Müller L (1932) Einige Schlangen von der Insel Milo. Vereinsberichte: “Isis”, München. Blätter für Aquarien- und Terrarien-Kunde 43: 130.

[B31] Nagy ZT Lawson R Joger U Wink M (2004) Molecular systematics of racers, whipsnakes and relatives (Reptilia: Colubridae) using mitochondrial and nuclear markers. Journal of Zoological Systematics and Evolutionary Research 42(3): 223–233. 10.1111/j.1439-0469.2004.00249.x

[B32] Nguyen LT Schmidt HA von Haeseler A Minh BQ (2015) IQ-TREE: A fast and effective stochastic algorithm for estimating maximum likelihood phylogenies. Molecular Biology and Evolution 32(1): 268–274. 10.1093/molbev/msu300 25371430 PMC4271533

[B33] Rozas J Ferrer-Mata A Sánchez-DelBarrio JC Guirao-Rico S Librado P Ramos-Onsins SE Sánchez-Gracia A (2017) DnaSP 6: DNA sequence polymorphism analysis of large datasets. Molecular Biology and Evolution 34(12): 3299–3302. 10.1093/molbev/msx248 29029172

[B34] Schätti B Stutz A Charvet C (2005) Morphologie, Verbreitung und Systematik der Schlanknatter *Platycepsnajadum* (Eichwald, 1831) (Reptilia: Squamata: Colubrinae). Revue Suisse de Zoologie 112: 573–625. 10.5962/bhl.part.80315

[B35] Schneider B (1979) Eine melanistische Schlanknatter, *Colubernajadumkalymnensis* n. subsp. (Colubridae, Serpentes), von der Insel Kalymnos (Dodekanes, Ägäis). Bonner Zoologische Beitrage 30(3–4): 383–387.

[B36] Schneider B (1983) Zur Herpetofauna der Inseln Kalymnos und Telentos (Dodekanes, Ägäis). Salamandra (Frankfurt) 19: 61–70.

[B37] Schweizer H (1935) Beitrag zur Reptilienfauna der Inselgruppe von Milos (Cycl.). Blätter für Aquarien- und Terrarienkunde 46: 8–15.

[B38] Sindaco R Venchi A Grieco C (2013) The Reptiles of the Western Palearctic, Volume 2: Annotated Checklist and Distributional Atlas of the Snakes of Europe, North Africa, Middle East and Central Asia, with an Update to Volume 1. Edizioni Belvedere, Latina, 543 pp.

[B39] Šmíd J Aghová T Velenská D Moravec J Balej P Naumov B Popgeorgiev G Üzüm N Avci A Jablonski D (2021) Quaternary range dynamics and taxonomy of the Mediterranean collared dwarf racer, *Platycepscollaris* (Squamata: Colubridae). Zoological Journal of the Linnean Society 193(2): 655–672. 10.1093/zoolinnean/zlaa151

[B40] Stephens M Smith NJ Donnelly P (2001) A new statistical method for haplotype reconstruction from population data. American Journal of Human Genetics 68(4): 978–989. 10.1086/319501 11254454 PMC1275651

[B41] Thanou E Kornilios P Lymberakis P Leaché AD (2020) Genomic and mitochondrial evidence of ancient isolations and extreme introgression in the four-lined snake. Current Zoology 66(1): 99–111. 10.1093/cz/zoz018 32467710 PMC7245017

[B42] Trifinopoulos J Nguyen LT von Haeseler A Minh BQ (2016) W-IQ-TREE: A fast online phylogenetic tool for maximum likelihood analysis. Nucleic Acids Research 44(W1): 232–235. 10.1093/nar/gkw256 PMC4987875 27084950

[B43] Tuniyev BS Orlov NL Ananjeva NB Aghasyan AL (2019) Snakes of the Caucasus: Taxonomic Diversity, Distribution, Conservation. KMK Scientific Press, St. Petersburg–Moscow, 276 pp.

[B44] Utiger U Schätti B (2004) Morphology and phylogenetic relationships of the Cyprus racer, *Hierophiscypriensis*, and the systematic status of *Colubergemonensisgyarosensis* Mertens (Reptilia: Squamata: Colubrinae). Revue Suisse de Zoologie 111: 225–238. 10.5962/bhl.part.80236

[B45] Werner F (1932) Eine neue Schlange aus dem Cycladen archipel. *Elapherechingeri* sp. n. Anzeiger der Akademie der Wissenschaften Wien 99: 232.

