# Supplementary material for: ﻿Endemic taxon or environmentally induced colour morph? Genetic insights into the insular subspecies of Platycepsnajadum (Serpentes, Colubridae) from Kalymnos, Greece
Source: Zookeys. 2025 Jul 4;1244:29–40. doi: 10.3897/zookeys.1244.152290 (PMC12254872; doi:10.3897/zookeys.1244.152290)
Supplement: Supplementary material 1 — Additional information [file zookeys-1244-029_article-152290__-s001.pdf]

A peer-reviewed open-access journal
ZooKeys
Launched to accelerate biodiversity research

Supplementary Information for

Endemic taxon or environmentally induced colour morph? Genetic insights into the insular subspecies of *Platyceps najadum* (Serpentes, Colubridae) from Kalymnos, Greece

Daniel Jablonski & Elias Tzoras

Table S1. A list of DNA loci, their corresponding primer sequences, sources, and the specific PCR conditions used for amplifications.

LOCUS	OR PRIMER SEQUENCE (5'-3')	PRIMER SOURCE	PCR CONDITIONS
**COI**	COI(+)deg1 F<br>AAGCTTCTGACTNCTACCACCNGC	Utiger et al. (2002)	94º (3'); 94º (60"), 57º (60"), 72º (60") x 35; 72º (10')
	COI(-)bdeg R<br>ATTATTGTTGCYGCTGTRAARTAGGCTCG	Utiger et al. (2002)	
**Cyt *b***	H16064 F<br>CTTTGGTTTACAAGAACAATGCTTTA	Burbrink et al. (2000)	94º (2'); 94º (20"), 48.5º (30"), 72º (75") x 37; 72º (10')
	L14910 R<br>GACCTGTGATMTGAAAACCAYCGTTGT	Burbrink et al. (2000)	
**C-mos**	S77 F<br>CATGGACTGGGATCAGTTATG	Lawson et al. (2005)	94º (7'); 94º (40"), 48º (30"), 72º (60") x 40; 72º (7')
	S78 R<br>CCTTGGGTGTGATTTTCTCACCT	Lawson et al. (2005)	
**NT3**	NT3-F3<br>F<br>ATATTTCTGGCTTTTCTCTGTGGC	Noonan and Chippindale (2006)	94°C (2'); 94º (40"), 48.5º (30"), 72º (60") x 40; 72º (7')
	NT3-R4<br>R<br>GCGTTTCATAAAAATATTGTTTGACC	Noonan and Chippindale (2006)	

Table S2. Material used for the genetic analysis.

			GenBank accession numbers				
**Species**	**Code**	**Country**	**COI**	**Cyt *b***	**C-mos**	**NT3**	**Source**
Platyceps collaris	HLMD J14	Jordan	-	-	AY486946	-	Nagy et al. (2004)
Platyceps collaris	DJ8200	Turkey	MT862578	MT862676	MT862603	MT862628	Šmíd et al. (2021a)
Platyceps elegantissimus	MHNG 2456.72	Saudi Arabia	AY039184	-	-	-	Schätti and Utiger (2001)
Platyceps florulentus	MHNG 2574.82	Ethiopia	AY039199	-	-	-	Schätti and Utiger (2001)
Platyceps florulentus	HLMD RA-3040	Egypt	-	AY486915	-	-	Nagy et al. (2004)
Platyceps gracilis	Gujarat	India	-	MZ020424	-	-	Deepak et al. (2021)
Platyceps josephi	ZSI-CZRC-V 6639	India	-	MZ020423	-	-	Deepak et al. (2021)
Platyceps karelini	IPMB J214	Uzbekistan	-	AY612003	-	-	Nagy et al. (2005)
Platyceps najadum	1881	Albania	PV656055	PV659844	PV659847	PV659852	This study
Platyceps najadum	R1399	Armenia	MT862588	MT862686	MT862613	MT862637	Šmíd et al. (2021a)
Platyceps najadum	383	Bosnia and Herzegovina	PV656053	PV659842	-	PV659850	This study
Platyceps najadum	NHMC 80.3.132.20 (sample 13307)	Greece	PV656057	PV659846	PV659849	PV659854	This study
Platyceps najadum	MHNG 2447.53	Greece	AY039179	-	-	-	Schätti and Utiger (2001)
Platyceps najadum	NHMC 80.3.132.19 (sample 13295)	Greece	PV656056	PV659845	PV659848	PV659853	This study
Platyceps najadum	MHNG 2642.69	Iran	AY897214	-	-	-	Schätti et al. (2005)
Platyceps najadum	539	Türkiye	PV656054	PV659843	-	PV659851	This study
Platyceps najadum	MHNG 2542.88	Türkiye	AY039166	-	-	-	Schätti and Utiger (2001)
Platyceps najadum	ZISP 27780	Armenia	-	AY486919	AY486943	-	Nagy et al. (2004)
Platyceps najadum atayevi	CAS 185188	Turkmenistan	-	AY486912	AY486943	-	Nagy et al. (2004)
Platyceps plinii	NCBS AQ-492	India	-	MZ020421	-	-	Deepak et al. (2021)
Platyceps rhodorachis	USNM:Herp:589593	Djibouti	MG700036	-	-	-	Gotte et al. (unpublished)

Platyceps rhodorachis	ZMUVAS24	Pakistan	MK941839	-	-	-	Ali et al. (2024)
Platyceps rhodorachis	-	China	-	MH043272	-	-	Weng (unpublished)
Platyceps rhodorachis	CAS 185035	Turkmenistan	-	AY486921	-	-	Nagy et al. (2004)
Platyceps rogersi	G3	Israel	MG566073	-	-	-	Sinaiko et al. (2018)
Platyceps rogersi	-	-	-	AY188041	-	-	Nagy et al. (2003)
Platyceps saharicus	SMNH<ISR>:17188	Israel	MF767367	-	-	-	Sinaiko et al. (2018)
***Platyceps* sp.**	USNM:Herp:581248	Iran	MG700031	-	-	-	Gotte et al. (unpublished)
***Platyceps* sp.**	SC-2016a	China	-	KT221058	-	-	Chen and Guo (unpublished)
Platyceps thomasi	OM137	Oman	-	MW204507	-	-	Šmíd et al. (2021b)
Platyceps variabilis	OM60	Oman	-	MW204506	-	-	Šmíd et al. (2021b)
Platyceps ventromaculatus	MHNG 2443.10	Pakistan	AY039174	-	-	-	Schätti and Utiger (2001)
Spalerosohis diadema	MHNG 2547.44	Yemen	AY039182	-	-	-	Schätti and Utiger (2001)
Spalerosohis diadema	MHNG 2414.68	Pakistan	AY039186	-	-	-	Schätti and Utiger (2001)
Spalerosohis diadema	CAS 220641	Egypt	-	AF471049	-	-	Lawson et al. (2005)
Spalerosohis diadema	RZ01	Iran	-	ON101678	-	-	Yadollahvandmiandoab et al. (2023)

Figure S1. DNA-investigated specimen of *Platyceps najadum kalymnensis* (NHMC 80.3.132.20) from Kalymnos Island, Greece. Photo by Daniel Jablonski.

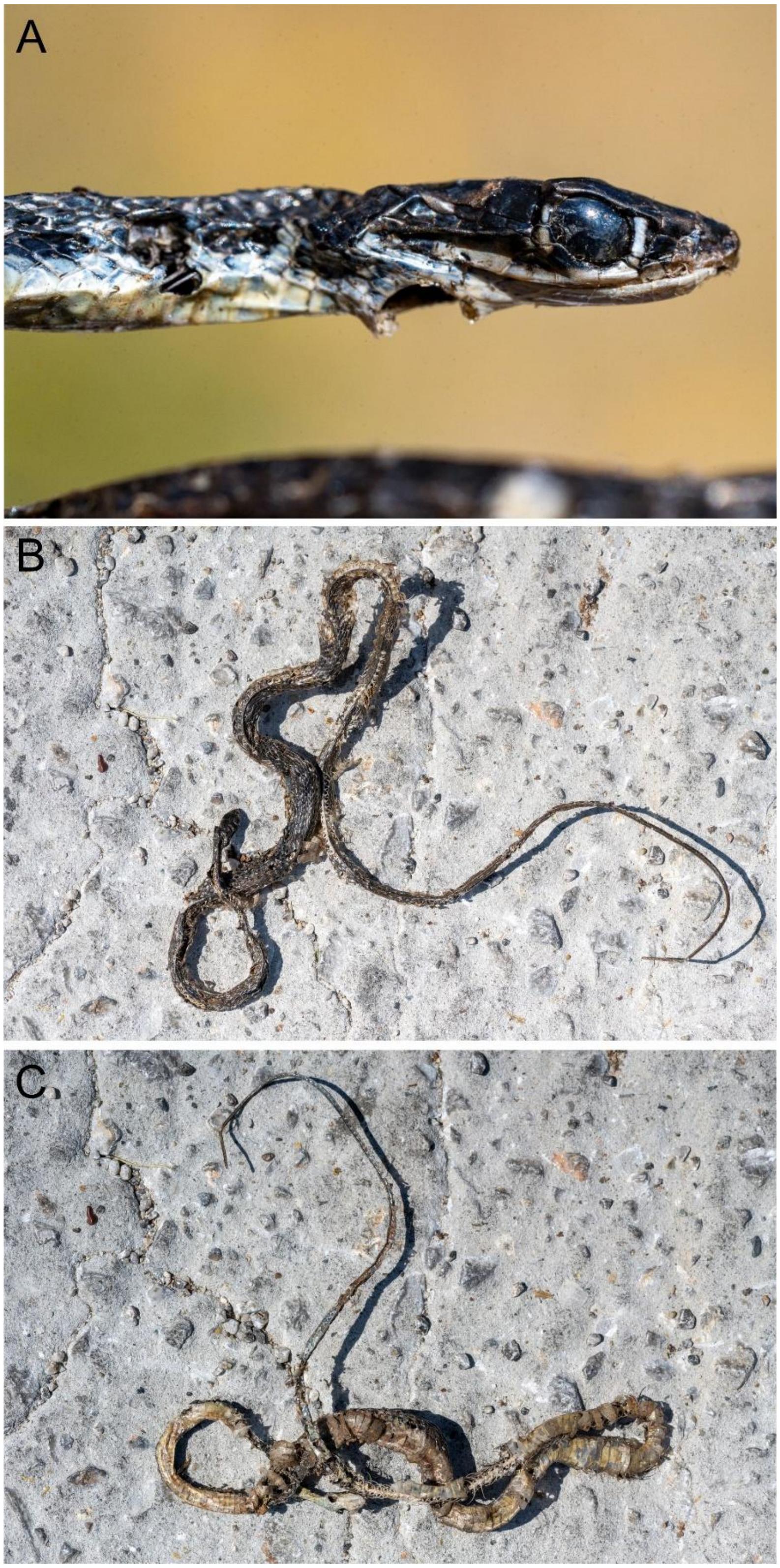

References to Supplementary information

Ali W, Bukhari SM, Ayub A, Qadir, G, Husain M, Masood M, Akhtar N, Alam H, Nawaz L, Javid A (2024) Molecular identification of herpetofauna from Pujab, Pakistan, using mtDNA genes. Journal of Wildlife and Biodiversity 8: 389–402. https://doi.org/10.5281/zenodo.12594174

Burbrink FT, Lawson R, Slowinski JB (2000) Molecular phylogeography of the North American rat snake (*Elaphe obsoleta*): a critique of the subspecies concept. Evolution 54: 2107–2114. https://doi.org/10.1554/0014-3820(2000)054[2107:mdpotp]2.0.co;2

Deepak V, Narayanan S, Mohapatra PP, Dutta SK, Melvinselvan G, Khan A, Mahlow K, Tillack F (2021) Revealing two centuries of confusion: new insights on nomenclature and systematic position of *Argyrogena fasciolata* (Shaw, 1802) (auctt.), with description of a new species from India (Reptilia: Squamata: Colubridae). Vertebrate Zoology 71: 253–316. https://doi.org/10.3897/vz.71.e64345

Lawson R, Slowinski JB, Crother BI, Burbrink FT (2005) Phylogeny of the Colubroidea (Serpentes): new evidence from mitochondrial and nuclear genes. Molecular Phylogenetics and Evolution 37: 581–601. https://doi.org/10.1016/j.ympev.2005.07.016

Nagy ZT, Joger U, Wink M, Glaw F, Vences M (2003) Multiple colonization of Madagascar and Socotra by colubrid snakes: evidence from nuclear and mitochondrial gene phylogenies. Proceedings of the Royal Society of London B 270: 2613–2621. https://doi.org/10.1098/rspb.2003.2547

Nagy ZT, Lawson R, Joger U, Wink M (2004) Molecular systematics of racers, whipsnakes and relatives (Reptilia: Colubridae) using mitochondrial and nuclear markers. Journal of Zoological Systematics and Evolutionary Research 42: 223–233. https://doi.org/10.1111/j.1439-0469.2004.00249.x

Nagy ZT, Vidal N, Vences M, Branch WR, Pauwels OSG, Wink M, Joger U (2005) Molecular systematics of African Colubroidea (Squamata: Serpentes). In: Huber BA, Sinclair BJ, Lampe KH (Eds) African Biodiversity, Springer, 221–228. https://doi.org/10.1007/0-387-24320-8_20

Noonan BP, Chippindale PT (2006) Dispersal and vicariance: The complex evolutionary history of boid snakes. Molecular Phylogenetics and Evolution 40(2): 347–358. https://doi.org/10.1016/j.ympev.2006.03.010

Schätti B, Utiger U (2001) *Hemerophis*, a new genus for *Zamenis socotrae* Günther, and a contribution to the phylogeny of Old World racers, whip snakes, and related genera (Reptilia: Squamata: Colubrinae). Revue Suisse de Zoologie 108: 919–948. https://doi.org/10.5962/bhl.part.80170

Sinaiko G, Magory-Cohen T, Meiri S, Dor R (2018) Taxonomic revision of Israeli snakes belonging to the *Platyceps rhodorachis* species complex (Reptilia: Squamata: Colubridae). Zootaxa. 4379: 301–346. https://doi.org/10.11646/zootaxa.4379.3.1

Šmíd J, Aghová T, Velenská D, Moravec J, Balej P, Naumov B, Popgeorgiev G, Üzüm N, Avci A, Jablonski D (2021a) Quaternary range dynamics and taxonomy of the Mediterranean collared dwarf racer, *Platyceps collaris* (Squamata: Colubridae). Zoological Journal of the Linnean Society 193: 655–672. https://doi.org/10.1093/zoolinnean/zlaa151

Šmíd J, Sindaco R, Shobrak M, Busais S, Tamar K, Aghová T, Simó-Riudalbas M, Tarroso P, Geniez P, Crochet PA, Els J, Burriel-Carranza B, Tejero-Cicuéndez H, Carranza S (2021b) Diversity patterns and evolutionary history of Arabian squamates. Journal of Biogeography 48: 1183–1199. https://doi.org/10.1111/jbi.14070

Utiger U, Helfenberger N, Schätti B, Schmidt C, Ruf M, Ziswiler V. 2002. Molecular systematics and phylogeny of Old World and New World ratsnakes, *Elaphe* auct., and related genera (Reptilia, Squamata, Colubridae). Russian Journal of Herpetology 9:105–124.

Yadollahvandmiandoab R, Koroiva R, Bashirichelkasari N, Mesquita DO (2023) Phylogenetic relationships and divergence times of the poorly known genus *Spalerosophis* (Serpentes: Colubridae). Organisms Diversity & Evolution 23: 415-423. https://doi.org/10.1007/s13127-022-00596-2
